# Insight into the population structure of hardhead silverside, *Atherinomorus stipes* (Teleostei: Atherinidae), in Belize and the Florida Keys using *nd2*

**DOI:** 10.1002/ece3.3457

**Published:** 2017-10-11

**Authors:** Chloe M. Nash, Michelle L. Kraczkowski, Barry Chernoff

**Affiliations:** ^1^ Biology Department Wesleyan University Middletown CT USA; ^2^ College of the Environment Wesleyan University Middletown CT USA; ^3^ Biology Department University of Saint Joseph West Hartford CT USA; ^4^ Department of Earth & Environmental Sciences Wesleyan University Middletown CT USA

**Keywords:** *Atherinomorus stipes*, Caribbean, *nd2*

## Abstract

Little is known about the natural history, biology, and population genetic structure of the Hardhead Silverside, *Atherinomorus stipes,* a small schooling fish found around islands throughout the Caribbean. Our field observations of *A. stipes* in the cays of Belize and the Florida Keys found that populations tend to be in close association with the shoreline in mangrove habitats. Due to this potential island‐based population structuring, *A. stipes* represents an ideal system to examine questions about gene flow and isolation by distance at different geographic scales. For this study, the mitochondrial gene *nd2* was amplified from 394 individuals collected from seven different Belizean Cays (*N* = 175) and eight different Floridian Keys (*N* = 219). Results show surprisingly high haplotype diversity both within and between island‐groups, as well as a high prevalence of unique haplotypes within each island population. The results are consistent with models that require gene flow among populations as well as in situ evolution of rare haplotypes. There was no evidence for an isolation by distance model. The *nd2* gene tree consists of two well‐supported monophyletic groups: a Belizean‐type clade and a Floridian‐type clade, indicating potential species‐level differentiation.

## 1 INTRODUCTION

The examination of genetic structuring of populations within marine systems provides insight into both historical and current evolutionary processes. However, the identification of genetic structuring within marine species is notoriously difficult due to the lack of clearly identifiable barriers to gene flow and dispersal commonly found within terrestrial systems. Early genetic studies on marine systems worked under the assumption that populations existed in a state of panmixia in the absence of extrinsic barriers, such as ocean currents and continental barriers (Avise, 2000; Taylor & Hellberg, 2006; D'Aloia, Bogdanowicz, Harrison, & Buston, 2014). However, studies have demonstrated clearly that other factors, such as habitat fragmentation and limited dispersal capabilities, may also act as reproductive barriers that restrict gene flow and result in subsequent isolation (Johnson & Black, 1991; Shulman & Bermingham, 1995; Fauvelot, Bernardi, & Planes, 2003; Gonzalez, Knutsen, & Jorde, 2016).

Dispersal capabilities, in particular, play a major role in shaping the genetic connectivity within marine species. Studies have shown that taxa with high dispersal capabilities maintain high levels of gene flow and express low levels of geographic structuring due to the homogenization of genetic diversity (Grant & Bowen, 1998; Beheregaray & Sunnucks, 2001; Manel, Schwartz, Luikart, & Taberlet, 2003; Gotoh, Chiba, Goto, Tamate, & Hanzawa, 2011; Manel & Holderegger, 2013). However, reduced dispersal capabilities minimize the amount of gene flow between populations, which may result in a nonrandom distribution of alleles (Templeton, Routman, & Phillips, 1995; Avise, 2000; Hanski, Erälahti, Kankare, Ovaskainen, & Sirén, 2004; Vekemans & Hardy, 2004).

Although it is extremely difficult to identify all of the factors that impact the dispersal of a species, an insight into the history, evolution, and phylogeography allows for a greater understanding of the complex population dynamics that drive the genetic structuring of populations (Tipton, Gignoux‐Wolfsohn, Stonebraker, & Chernoff, 2011). This scientific perspective is essential for proper conservation management of marine taxa, especially for organisms that inhabit vulnerable environments undergoing rapid disturbance, fragmentation, and destruction.

**Figure 1:**
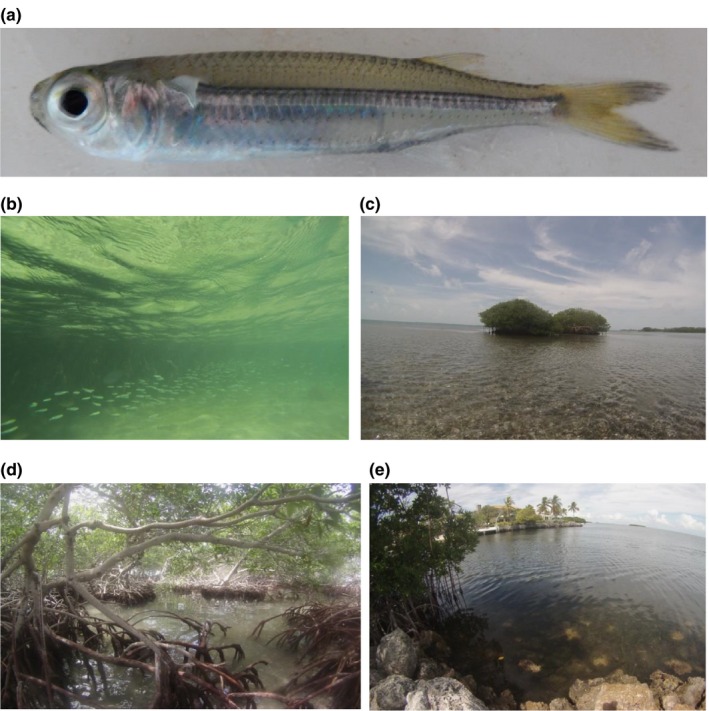
*Atherinomorus stipes* and selected habitats. Photographs of (a) an individual *A. stipes* from Belize, (b) a school of *A. stipes* in Belize, (c) Blue Range Cay sample site in Belize, (d) Stewart Cay sample site in Belize, and (e) Plantation Key sample site in Florida. All photographs were taken by Chloe Nash

The aim of this study is to analyze the phylogeography of the Hardhead Silverside, *Atherinomorus stipes* (Müller & Troschel, 1848; Figure 1a), within and between island‐groups found off the coast of Belize and along the Florida Keys. *Atherinomorus stipes* belongs to the family Atherinidae, commonly known as true silversides, within the order Atheriniformes, which consist primarily of ecologically important surface foragers found throughout temperate and tropical regions (Bloom, Unmack, Gosztonyi, Piller, & Lovejoy, 2012). This species, a planktivore that feeds equally in sea grass and mangrove habitats (Vaslet et al., 2015), is one of the most abundant fishes found in close association with mangrove communities throughout the Caribbean (Vaslet, Bouchon‐Navaro, Charrier, Louis, & Bouchon, 2010; Vaslet, Bouchon‐Navaro, Louis, & Bouchon, 2010). Despite their ubiquity, *A. stipes* has received very little scientific attention. Their phylogenetic placement, both within the family Atherinidae and within the Atheriniformes, has been debated for many years and remains unresolved (Dyer & Chernoff, 1996; Aarn & Ivantsoff, 1997; Sparks & Smith, 2004; Bloom et al., 2012; Near et al., 2012; Betancur‐R et al., 2013; Sasaki & Kimura, 2014; Campanella et al., 2015).

We examine the population structure of *A. stipes* in two island‐groups: the Belizean Cays and the Florida Keys. The close association of *A. stipes* to mangrove shores is ideal for examining population structuring within and between island‐groups because of the potential for the restriction of gene flow due to habitat heterogeneity, fragmentation of suitable habitat, and distance among islands. We sequence the mitochondrial gene *nd2* in order to address the following questions: (i) are the Belize Cays and Florida Keys island‐groups genetically homogeneous and (ii) are populations within each island‐group homogeneous? We predict that populations of *A. stipes* exhibit genetic structuring both within and between the island‐groups. We also predict that the isolation by distance (IBD) model will explain genetic divergence in relation to geographic distance.

## 2 MATERIALS AND METHODS

### 2.1 Sample collection

**Figure 2:**
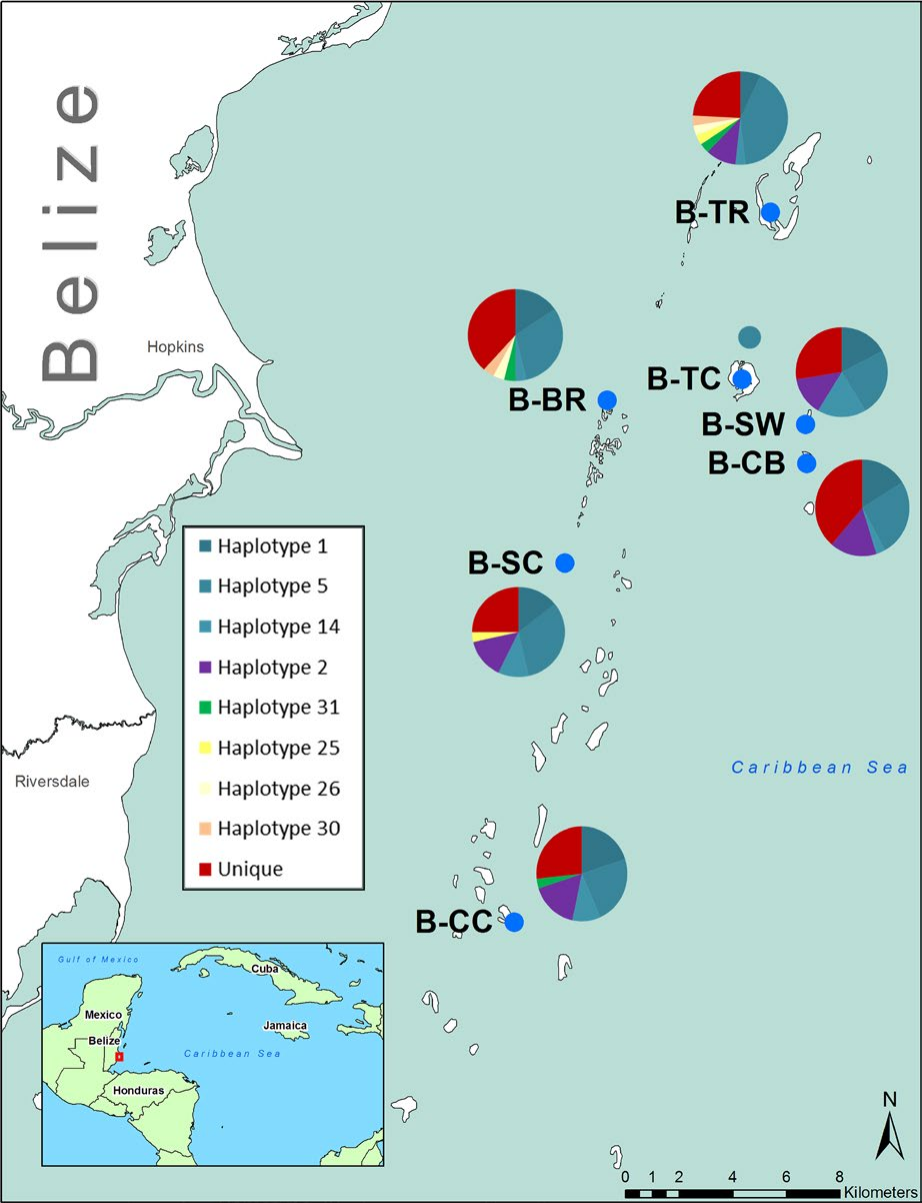
Haplotype frequency within Belizean populations. Sampling locations are indicated by the blue dot and accompanied by the site code and a pie graph of their haplotypes. Pie graphs display the frequency of haplotypes of *nd2* within each population. Each pie graph is color coded to display the haplotypes found in that population. The blue portions of each graph represent haplotypes that were shared among all island populations, and the red portion of each pie graph indicates the proportion of population‐specific haplotypes. Other colors are haplotypes shared among some populations. Code designations can be found in Tables 1 and A1

**Figure 3:**
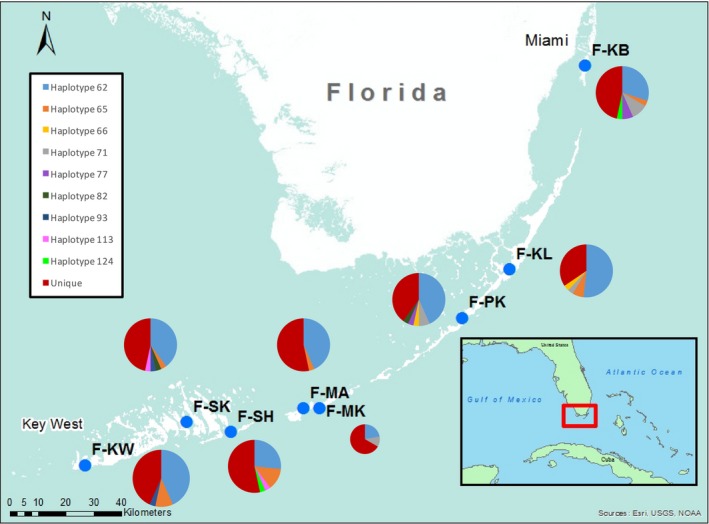
Haplotype frequency within Floridian populations. Sampling locations are indicated by the blue dot and accompanied by the site code and a pie graph of their haplotypes. Pie graphs display the frequency of haplotypes of *nd2* within each population. Each pie graph is color coded to display the haplotypes found in that population. The blue portions of each graph represent the universal haplotype, and the red portion of each pie graph indicates the proportion of population‐specific haplotypes. Other colors are haplotypes shared among some populations. Code designations can be found in Tables 1 and A1

The range of *A. stipes* has been described as Caribbean‐wide, but an explicit habitat characterization is lacking (Chernoff, 2002; Vaslet, Bouchon‐Navaro, Charrier, et al., 2010; Vaslet, Bouchon‐Navaro, Louis, et al., 2010). Based on observations made in the field, we note that the ideal habitat for *A. stipes* was within close proximity to mangrove roots in combination with a sandy bottom substrate and the presence of turtle grass (Figure 1). Individual islands in Belize and Florida were chosen for sampling based on the presence of this described habitat, their geographic distance to other sampled islands, and the accessibility for collection (Figures 2 and 3); the latitude and longitude of each sampling location is listed in Table A1.

A total of 394 individuals of *A. stipes* were collected for this study, amounting to approximately 25–30 individuals per island population. Of these, 393 were successfully sequenced. We collected Belizean samples (Belize Dept. of Environment 000011763) in July 2014, with the exception of B‐CB1_A01, and all Floridian samples in August 2015. All specimens were caught by 2‐m seine net with 3.2‐mm mesh. Individual B‐CB1_A01 was received as a voucher specimen from the University of Kansas Biodiversity Institute (Voucher Specimen #USNM 349215).

Caudal fin clips were stored in individual tubes in either 95% ethanol (Belizean samples) or Chaos Buffer (Floridian samples; Crawford & Oleksiak, unpublished) until DNA extraction. Voucher specimens were initially stored in formalin and then transferred to a 70% ethanol solution for long‐term storage for future morphological studies. Individuals were grouped by island population, and each individual was given a coded ID consisting of: (Island‐group Name)‐(Island Population Name)_(Individual ID Number). For example, sample B‐CB_A01 represents a sample from the Belizean island‐group, the Carrie Bow Cay island population, and was individual A01. All location codes are in Table 1, and the corresponding location names are in Table A1.

**Table 1:** Population polymorphism statistics

Location code	Sample size	Total number of haplotypes	Number of unique haplotypes	Haplotype diversity (Hd)	Nucleotide diversity (π)	Average no. of bp differences between haplotypes
Belize						
B‐CB	31	16	12	0.897	0.00257	3.049
B‐SW	29	12	8	0.884	0.00283	3.365
B‐TC	1	1	0	–	–	–
B‐TR	29	14	6	0.825	0.00302	3.581
B‐BR	26	16	10	0.895	0.00355	4.212
B‐CC	30	13	8	0.887	0.00253	3.005
B‐SC	29	11	6	0.862	0.00312	3.709
Florida Keys						
F‐KL	29	14	10	0.739	0.00162	1.92611
F‐PK	30	17	12	0.818	0.00159	1.88736
F‐MK	9	8	6	0.972	0.00304	3.61111
F‐KW	30	11	8	0.798	0.00148	1.75632
F‐SK	28	17	13	0.825	0.00178	2.10847
F‐SH	28	18	15	0.910	0.00237	2.80952
F‐MA	30	18	16	0.821	0.00173	2.04828
F‐KB	30	18	13	0.906	0.00190	2.25977

The table displays the sample size, total number of haplotypes, the total number of population‐specific haplotypes, the haplotype diversity (Hd), the nucleotide diversity (π), and the average number of mutations between haplotypes within each island population sample. The sample sizes reflect successfully sequenced individuals minus four highly differentiated Florida fish.

### 2.2 Molecular work

The extraction of DNA from the individual fin clips occurred within 2 weeks after collection. *Belize*: DNA from the caudal fin clips collected in the Belizean Cays was extracted using the DNeasy Blood and Tissue Kit: QIAGEN Sciences, MD, USA. The provided protocol was followed with the exception of the last step, in which the total DNA yield was increased to 200 μl by repeating the final 100 μl elution step. *Florida*: DNA from the caudal fin clips collected from the Florida Keys was extracted following the protocol for gDNA for Genome Based Sequencing (GBS; Crawford & Oleksiak, unpublished). Approximately 100 μl of extracted DNA was produced per sample. Final DNA concentration for all samples was determined on a Thermo Scientific NanoDrop™ ND‐2000 1‐position spectrophotometer.

The mitochondrial gene *nd2* was amplified and analyzed for this study. Mitochondrial genes are useful in analyzing the matrilineal relationships between populations and closely related species due to their high variability within species (Avise, 2000). A ~1,200 base‐pair (bp) fragment of *nd2* was amplified using the GLN and ASN primers obtained from Kocher et al., 1995. PCR parameters followed the protocol of Tipton et al. (2011). Seven μl of PCR product mixed with 1 μl of Gel Loading Dye was run at 100 V for 30 min in a 1.5% agarose gel with 5 μl of SYBRsafe (Invitrogen). Samples with a visible band ~1,200 bp in length were purified in each primer direction following the Exo‐AP PCR product purification protocol described by the DNA Analysis Facility on Science Hill at Yale University for Standard Service Sequencing; all samples were shipped and sequenced at this facility. The forward and reverse sequences were aligned using ClustalW multiple alignment in BioEdit v7.1.7 (Hall, 1999) and curated by hand based on chromatograms viewed in FinchTV 1.4 (Geospiza, Inc.).

Two sequences of *nd2* from *A. stipes* collected in Barbados were accessed from GenBank (GenBank #KC736458.1, GenBank #KC736457.1; Bloom, Weir, Piller, & Lovejoy, 2013). Sequences from the following taxa were used as out‐groups: *Atherinomorus lacunosus* (GenBank #KJ667868; Stelbrink, Stöger, Hadiaty, Schliewen, & Herder, 2014) and *Hypoatherina tsurugae* (GenBank #AP004420.1; Miya et al., 2003). The curated sequences were deposited in GenBank under accession numbers MF924405–MF924566.

### 2.3 Phylogenetic analyses

The model of best fit of sequence evolution for all haplotypes was GTR + I based on AIC and BIC indices (jModelTest v2.1.4; Guindon & Gascuel, 2003; Darriba, Taboada, Doallo, & Posada, 2012). This model was used to produce maximum‐likelihood (ML) and Bayesian phylogenetic trees. The Bayesian analysis was performed in MrBayes 3.2.6 (Ronquist et al., 2012) using a Markov Chain Monte Carlo (MCMC) method sampled every 100 generations for a total of 5,350,000 generations. A 50% majority rule consensus tree was generated after a burn in of the first 25% of sampled generations. A phylogenetic tree based on maximum‐parsimony (MP) assumptions was also generated with the same dataset. ML and MP phylogenetic trees were created in MEGA6 (Tamura, Stecher, Peterson, Filipski, & Kumar, 2013). The final MP tree is a consensus of the three most equally parsimonious trees, and the final ML tree is the tree with the highest supported nodes under the maximum‐likelihood framework. All phylogenetic trees were visualized in FigTree v1.4.2 (http://tree.bio.ed.ac.uk/software/figtree/) and annotated using the R package “ggtree” (Yu, Smith, Zhu, Guan, & Lam, 2016). TCS v1.21 (Clement, Posada, & Crandall, 2000) was used to create a statistical parsimony haplotype network with a connection limit set to 95% for both the Floridian and Belizean island‐groups.

It should be noted that four individuals from the Florida Keys (F‐SK_A301, F‐SK_A302, F‐SH_A309, F‐SH_A317) exhibited highly differentiated haplotypes from all other Floridian individuals. As outliers, they were removed from the Florida dataset used in all of the following analyses. DnaSP v5.10.01 (Librado & Rozas, 2009) was used to calculate haplotype counts, nucleotide diversity (π), haplotype diversity (Hd), and the average number of mutations between haplotypes for each island population of *A. stipes* (Nei & Kumar, 2000). The average number of pairwise differences between populations was calculated in Arlequin 3.5.1.2 (Excoffier & Lischer, 2010). The following tests of neutrality were also executed in Arlequin: Tajima's D, Fu's *F*_S,_ Fu and Li's D*, and Fu and Li's F* (Tajima, Misawa, & Innan, 1998; Fu, 1997; Fu & Li, 1993).

Analyses of molecular variance (AMOVA) was conducted in Arlequin 3.5.1.2 (Excoffier & Lischer, 2010). AMOVAs were generated for the following group structures: among island populations within the Belize island‐group, among island populations within the Florida Keys island‐group, between island populations on the Gulf of Mexico versus the Atlantic Ocean side of the Florida Keys island‐group, and between the Belize and Florida Keys island‐groups. Pairwise distances (*F*_ST_) between individual island populations within each island‐group were also generated in Arlequin.

When genetic structuring is observed, the role of the IBD model of evolution can be assessed (Wright, 1943). An analysis of IBD allows for an evaluation of both dispersal and the amount of gene flow that is occurring between populations (Puebla, Bermingham, & Guichard, 2009). Evidence for the IBD model of evolution within each island‐group was tested by performing a linear regression analysis, using both log and standardized scale transformations in the R Stats Package (R Core Team 2000) and a Mantel permutation test in Arlequin using 10,000 randomized replicates to calculate statistical significance (Mantel, 1967). The linear regression analysis used the average number of pairwise differences between populations as the measure of genetic distance, and the Mantel permutation tests utilized population pairwise *F*_ST_ values. Both the linear regression analysis and the Mantel permutation test used the Euclidean geographic distance between populations, calculated in ArcMap v10.3.1 (ESRI, Redlands, CA, USA).

## 3 RESULTS

### 3.1 Genetic diversity and differentiation

About 1,187 base pairs for the *nd2* gene were successfully determined and aligned for 394 individuals of *A. stipes*. There were 178 variable sites among all individuals, and 94 of these were parsimony informative sites.On average there were 3,487 nucleotide differences among haplotypes from the Belizean Cays and 2,301 nucleotide differences among haplotypes from the Florida Keys. In Belize, the average Hd was 0.875 (range: 0.825 ≤ Hd ≤ 0.897), and the average nucleotide diversity (π) was 0.00294 (0.00253 ≤ π ≤ 0.00355; Table 1). Within the Florida Keys, the average value of Hd was 0.849 (0.739 ≤ Hd ≤ 0.972), and the value of π was 0.00194 (0.00148 ≤ π ≤ 0.00304; Table 1). Overall, Hd, π, and the average number of mutations between haplotypes were greater within the Belizean island‐group than within the Floridian island‐group.

A total of 58 haplotypes were observed within the Belizean island‐group and 104 haplotypes within the Floridian island‐group. Among the 58 haplotypes identified in the Belizean island‐group, eight haplotypes were shared by more than one population as follows: (i) three haplotypes (1, 5, and 14) were “universal,” defined as being shared among all populations; (ii) haplotype 2 was shared among five populations; (iii) haplotype 31 was found in three populations; and (iv) and three haplotypes (25, 26, and 30) were found in two populations (Figure 2). Only one individual was captured at location B‐TC, and it possessed one of the “universal” haplotypes (haplotype 5). The remaining 50 haplotypes were classified as “unique,” which we defined as being a population‐specific haplotype^1^ (Figure 2). The total number of haplotypes within each population ranged from 11 to 16, with six to 12 of these haplotypes deemed “unique” to a population (Table 1). Approximately 50% of individuals within each population expressed one of the three universal haplotypes. Approximately 20–30% of individuals within each population exhibited a “unique” haplotype (Figure 2).

Among the 104 total haplotypes identified in Florida, nine were distributed as follows: (i) haplotype 62 was “universal”; (ii) haplotype 65 was shared among six island populations; (iii) haplotype 66 was observed among four island populations; and (iv) six haplotypes (haplotypes 71, 77, 82, 93, 113, and 124) were found in two populations. The remaining 95 haplotypes were “unique” (Figure 3; Table 1). The total number of haplotypes observed in each population ranged from eight to 18. The population from the Atlantic side of Marathon Key (F‐MK) consisted of only nine individuals that possessed eight haplotypes. Of these, six were “unique” to F‐MK. In all other sampled populations, which consisted of approximately 30 individuals each, the fewest number of total haplotypes observed was 11, and the number of “unique” haplotypes ranged from eight to 16 (Table 1). The “universal” haplotype was found in 22–52% of individuals within all populations (Figure 3). The percentage of individuals with “unique” haplotypes within each population ranged between 35% and 67% (Figure 3).

All tests of neutrality produced negative values for all populations (Table A2). Values of Tajima's D for all Florida populations, with exception of F‐MK, were statistically significant (*p < *.05), while none of the Belizean populations were found to be statistically significant (Table A2). Fu's *F*_S_ was statistically significant for four populations from Belize (*p < *.05) and all populations from the Florida Keys (*p < *.02; Table A2). Fu and Li's F* and D* statistics had congruent patterns of significance in each island‐group, with three Belizean populations and five Floridian populations having significant values (*p < *.05 for both; Table A2).

The variation between the Belizean and Floridian island‐groups was found to be highly significant (*p < *.00001; Table 2). Additionally, a global AMOVA comparing the populations within Belize indicated that the among‐groups variance was also highly significant (*p < *.00001; Table 2). However, the sources of variation attributable to the “among populations within groups” and “within populations” categories were not significant (Table 2). Within Florida, the variation among and within populations was highly significant (*p *<* *.00001 and *p *=* *.02444, respectively; Table 2). There were no significant differences (*p *>* *.078) between populations on the Gulf of Mexico side of the Florida Keys versus those populations on the Atlantic side (Table 2).

**Table 2:** Global AMOVAs

	Source of variation	Variance components	Percentage of variation	*p*‐Value
Within Belize	Among groups	0.06351	3.6773	<.00001***
	Within populations	1.73295	100.33615	.90616^NS^
Within Florida Keys	Among groups	0.04986	4.56396	<.00001***
	Within populations	1.08302	99.14229	.02444*
Belize vs. Florida Keys	Among groups	23.5395	88.82084	<.00001***
	Within populations	2.99976	11.3189	<.00001***
Florida: Gulf vs. Atlantic	Among groups	−0.00546	−0.5	.97165 ± .00578^NS^
	Within populations	1.08302	99.37	.08602 ± .00879^NS^

Summary of Global analyses of molecular variance (AMOVA) statistics with groups defined as all populations within Belize, all populations within the Florida Keys, the Belize and Florida Keys island‐group, and populations located on the Gulf of Mexico side of the Florida Keys against populations located on the Atlantic side of the Florida Keys. All AMOVAs were generated in Arlequin. The samples reflect successfully sequenced individuals minus four highly differentiated Florida fish. Significance is indicated as follows: *p <* *.05, ***p <* *.001, ^NS^ *p *>* *.05.

### 3.2 Relationships among haplotypes

**Figure 4:**
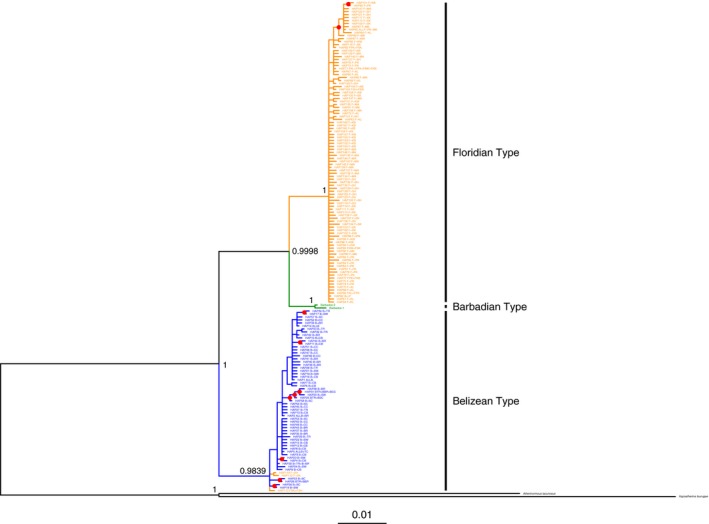
50% majority rule consensus tree of haplotypes of *nd2* in *Atherinomorus stipes*. 50% majority rule tree Bayesian tree generated in MrBayes. This tree topology is corroborated by maximum‐likelihood, maximum‐parsimony, and Bayesian analyses. Haplotypes from Florida are indicated in orange, haplotypes from Belize in red, and haplotypes from Barbados in blue. Interior nodes with posterior probability (pp) values >.90 are indicated with red dots. Exterior nodes display exact posterior probability values

The phylogenetic analyses (ML, MP, and Bayesian) corroborated a tree topology identifying two distinct, well‐supported, monophyletic groups (Figure 4): (i) a clade of Belizean‐type haplotypes; and (ii) a clade of Floridian‐type haplotypes plus Barbadian‐type haplotypes (ML bootstrap value bv = 0.988, MP bv = 1, posterior probability pp = .9839; ML bv = 0.999, MP bv = 1, pp = .9998 respectively; Figure 4). The genetic divergence between these two major clades was approximately 4.5% (Figure 4). Neither clade exhibited evidence of spatial clustering of haplotypes by population.

Within the Floridian‐type/Barbadian‐type clade, haplotypes from the Florida Keys and haplotypes from Barbados formed two separate, well‐supported monophyletic groups (Figure 4). The genetic divergence between the Floridian‐type and Barbadian‐type haplotypes was approximately 2.3%. The Belizean‐type haplotypes formed a well‐supported monophyletic group sister to the Floridian‐type/Barbadian‐type clade (Figure 4). Surprisingly, four fish sampled from Floridian populations had haplotypes that were highly divergent from all other Floridian fish and were nested within the Belizean‐type haplotype clade (Figure 4).

**Figure 5:**
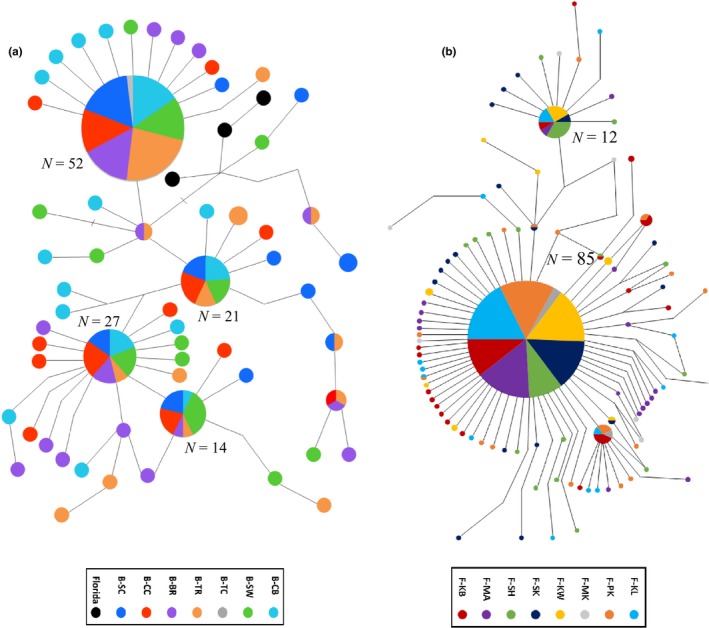
Haplotype network of Belizean‐type and Floridian‐type haplotypes. Ninety‐five percent statistical parsimony network showing the relationships between haplotypes of *nd2* for (a) Belizean populations and (b) Floridian populations (Right) of *Atherinomorus stipes* (generated in TCS). Each circle represents a single haplotype. The size of the circle indicates the frequency of the haplotype, and the exact number of individuals is shown for the major haplotypes. Each line represents a single base‐pair (bp) mutation, while corner kinks and hash marks indicate an additional bp mutation. Colors (are not shared among Belizean and Floridian populations) indicate the populations where haplotypes were observed. The black circles represent the highly differentiated Floridian individuals that are able to connect in the Belize haplotype, but not the Florida network. Code designations can be found in Tables 1 and A1

The statistical parsimony haplotype networks for Belizean‐type haplotypes and Floridian‐type haplotypes differed greatly in their structures. The Belizean‐type haplotype network exhibited a more complex structure with four major “universal” haplotypes found in varying frequencies (*N* = 14–52; Figure 5a). These four “universal” haplotypes were separated by one or two base‐pair substitutions from the majority of minor haplotypes, with the maximum number of steps from a major a haplotype being seven (Figure 5a). There was no spatial clustering of haplotypes by population. Three haplotypes from the four highly differentiated individuals collected in Florida did not connect with the main Floridian haplotype network. Instead, they connected to the Belizean haplotype network (denoted as black circles in Figure 5a). These three haplotypes differed by four‐to‐six base‐pair substitutions from two of the major Belizean haplotypes (Figure 5a).

In contrast, the Floridian‐type haplotype network exhibited a classic “starburst” pattern (Figure 5b). Starbursts consist of a single common haplotype with numerous minor haplotypes that are one or two base pairs removed from this common haplotype (Shields & Gust, 1995; Grant & Bowen, 1998; Avise, 2000). The common haplotype, termed “universal” in this study, found in Florida was observed in 85 individuals among all Florida populations in nearly equal proportion (Figures 3 and 5b). The vast majority of minor haplotypes, which were predominately classified as “unique,” were only observed at a frequency of one or two total individuals.

### 3.3 Isolation by distance

Linear regression analyses using log and standardized axes resulted in nonsignificant correlations within both the Belizean and Floridian island‐groups (adjusted *r* ^2^ = −.04291; *p = *.6786 and adjusted *r* ^2^ = −.01987; *p *=* *.4973, respectively). Mantel tests comparing the pairwise *F*_ST_ to the Euclidian geographic distance between populations within the Belizean and Floridian island‐groups revealed that there was only a slightly significant correlation within the Belizean island‐group (*p = *.0475).

## 4 DISCUSSION

Haplotypes of *nd2* from *A. stipes* were highly divergent (*ca*. 4.5%) between the Belize Cays and Florida Keys island‐groups. The two specimens from Barbados, the type locality of *A. stipes*, formed the sister group to the Floridian clade (Figure 4). The divergence between the sister lineages was 2.3%. Although the clades of silversides from Florida/Barbados and Belize were reciprocally monophyletic, four individuals from Florida had haplotypes that placed them within the Belizean clade.

The following genetic characteristics were observed within both island‐groups: (i) high levels of haplotype diversity (Hd) and low levels of nucleotide diversity (π); (ii) high proportions of rare “unique” haplotypes associated with particular island populations; and (iii) significant molecular variation among populations. Combined, these genetic characteristics are consistent with island‐group populations that have experienced a recent rapid expansion with subsequent accumulation of novel mutations after one or more bottleneck events (Avise, Neigel, & Arnold, 1984; Grant & Bowen, 1998; Tipton et al., 2011). The haplotype divergence patterns among populations within island‐groups were very different. The Floridian island‐group exhibits the well‐defined starburst pattern (Figure 5) with most haplotypes differing from the most‐common haplotype by a single mutation. In contrast, the Belizean clade had four common haplotypes forming connected starbursts. The four Floridian specimens that fell within the Belizean clade differed minimally from two common haplotypes by four mutations (Figure 5). Here we discuss possible phylogeographic scenarios in an attempt to explain the observed structural motifs of *nd2* in this species.

### 4.1 Potential speciation

The most striking result of this study was the surprisingly large degree of divergence between the major haplotypes of *nd2* in Belize and the Florida Keys. These island‐group clades were found to be distinct and highly significant (AMOVA; *p < *.00001) with a genetic divergence of 4.5% (Figure 4). Additionally, the Florida and Barbados populations differed by 2.3%. These levels of intraspecific divergence are considered to be relatively large and could indicate that *A. stipes* from within each of these island‐groups represent independent evolutionary lineages (Gomes, Pessali, Sales, Pompeu, & Carvalho, 2015).

Evolutionary forces acting on isolated gene pools can result in rapid genetic differentiation and potential speciation (Barraclough, 1998; Puebla et al., 2009; O'Leary et al., 2016). The varying degrees of differentiation among the haplotypes from Belize, Florida Keys, and Barbados suggest that there was restricted gene flow among certain island‐groups. Although the type locality of *A. stipes* (Müller & Troschel, 1848) is Barbados, confidence in the application of this species name requires a comprehensive analysis across its geographic range.

While the use of a single mitochondrial gene to analyze population structure is limited due to potential discordance with other gene trees, it represents an important starting point for examining the phylogeographic patterns of this species (Degnan & Rosenberg, 2009). The identification of species boundaries based upon morphology can underestimate biodiversity throughout the marine realm (Knowlton, 2000). Genetic studies of atheriniform fishes have provided many excellent examples of the clarification of clades and species boundaries (e.g., *Atherina boyeri*—Klossa‐Kilia, Papasotiropoulos, Tryfonopoulos, Alahiotis, & Kilias, 2007; *Menidia conchorum*—O'Leary et al., 2016). The evolution of independent genetic lineages that create cryptic biodiversity has important conservation implications because current management practices may not protect each discrete, genetic stock (Beheregaray & Sunnucks, 2001).

### 4.2 Neutrality tests

Tests of evolutionary neutrality indicate that Belizean and Floridian populations evolved, at least recently, under random and nonrandom (neutral) processes, respectively. The Floridian populations exhibited a classic starburst pattern haplotype network (Figure 5) and had highly significant negative values of Tajima's D, Fu and Li's D*, and Fu and Li's F* (Table A2). These attributes suggest populations were evolving under nonrandom evolutionary processes, with the most plausible scenario being a recent demographic crash, such as a population bottleneck, followed by a rapid expansion in population size (Grant & Bowen, 1998; Avise, 2000; Depaulis, Mousset, & Veuille, 2003; Venkatesan, Westbrook, Hauer, & Rasgon, 2007; Tipton et al., 2011).

The haplotypes of Belizean populations exhibited a complex pattern that included several connected starbursts (Figure 5). This pattern is similar to the haplotype network observed in several populations of *A. endrachtensis* within isolated marine lakes in Palau (Gotoh et al., 2011). The nonsignificant values of Tajima's D, Fu's *F*_S_, Fu and Li's D*, and Fu and Li's F* (Table A2) for most of the Belizean populations suggest that *nd2* was evolving neutrally and did not depart from the genetic drift mutation equilibrium (Tajima et al., 1998; Fu, 1997; Fu & Li, 1993; Gotoh et al., 2011).

A recent bottleneck event or demographic crash results in significant, negative values of Tajima's D and Fu's *F*_S_ due to the excess of rare alleles that arise within the population during its recovery and subsequent expansion (Depaulis et al., 2003). Because Tajima's D and Fu's *F*_S_ have greater statistical power for detecting more recent events, these statistics may not illuminate older demographic crashes (Depaulis et al., 2003). This could explain the observed discrepancy between the neutrality tests for the Belizean and Floridian island‐groups.

Additionally, the degree of habitat disturbance or destruction can markedly affect the genetic structure of fish populations, resulting in deviations from neutral evolutionary processes (Shulman & Bermingham, 1995; Fauvelot et al., 2003; Gonzalez et al., 2016). The mangrove habitat found along the Florida Keys has been highly disturbed by coastal development. The degraded and discontinuous state of these habitats may serve to fragment Floridian populations. This is in stark contrast to the majority of observed mangrove habitats within the protected waters off the coast of Belize.

### 4.3 Population‐level differences and gene flow within island‐groups

Within each island‐group, populations differed significantly from each other and had a high percentage of “unique” haplotypes (Figures 2 and 3). “Unique” haplotypes comprised between 20% to almost 70% of the total haplotypes within each island population (Figures 2 and 3). Nonetheless, each population contained relatively high percentages of common haplotypes.

There are several ways that the pattern of genetic similarities and dissimilarities among populations within island‐groups could have evolved. There are two possible parsimonious explanations: (i) limited gene flow among populations, where rare, “unique” haplotypes result from in situ evolution over time diminishing the frequency of the common haplotypes; and (ii) moderate or higher amounts of gene flow among populations, where rare haplotypes are not necessarily “unique.” Both of these assume that the common haplotype is relatively older than the rarer haplotypes, but differ in their interpretation of the proportion of “unique” haplotypes within each population. The classification of a haplotype as “unique” is based upon sampling. In this case, our sample sizes (25.9 individuals per population on average) may not have been sufficiently large to detect haplotypes in very low frequencies, and therefore, overestimated the uniqueness of haplotypes and the degree of isolation among populations. Common haplotypes have a higher probability of being exchanged among populations compared to rarer haplotypes and, hence, explain the prevalence of common haplotypes in many populations. Our data are not sufficient to reject either of these hypotheses.

Some of the results indicate more isolation of populations than would be expected from the second hypothesis. The genetic compositions of the samples from the two Marathon Key, Fla., sites suggest relative isolation of these geographically proximate localities. The sample taken on the Atlantic side (F‐MK) had only nine individuals that had eight different haplotypes for which six were “unique.” The Gulf of Mexico sample from Marathon Key (F‐MA) had 30 individuals with 18 haplotypes of which 16 were “unique.” Despite the fact that they were collected on opposite sides of the same key (5.8 km between the sample sites), none of the “unique” haplotypes from the small Atlantic side sample were found in the larger sample from the Gulf side of Marathon Key (no Atlantic vs. Gulf of Mexico effect was found among the Floridian samples). That said, we do note that the preferred habitat of *A. stipes*, mangroves and sea grass beds, around the shoreline of the key is not continuous. Similarly, in Belize, South Water Cay (*N* = 29) and Carrie Bow Cay (*N* = 31) differed by 21 “unique” haplotypes despite being separated by only 1.4 km of open water.

To what extent has gene flow been occurring among islands? Is the gene flow historic or contemporary? Despite the genetic heterogeneity among populations, the data do not suggest that populations have been completely isolated. The results are consistent with the inference that there has been gene flow among populations because of the relative frequencies of the common haplotypes. The common haplotypes are most parsimoniously interpreted as older in origin than the rare, “unique” haplotypes. The common haplotypes may represent ancestral or founder haplotypes (Templeton & Sing, 1993; Crandall, 1996; Avise, 2000; Gotoh et al., 2011; Tipton et al., 2011). The life history characteristics of *A. stipes* may serve to fragment this species and reduce gene flow (discussed below). Fragmented populations tend to evolve more rapidly due to higher levels of genetic drift (Barraclough, 1998; Puebla et al., 2009; O'Leary et al., 2016), and thereby explain the very high percentages of “unique” haplotypes within populations.

### 4.4 Isolation by distance

Isolation by distance predicts that genetic similarity is inversely proportional to geographic distance among populations. Evidence for IBD in marine systems is relatively rare and can vary widely due to the spatial scales of the sampling locations, the population density, and the dispersal capabilities of marine species (Puebla et al., 2009). The regression analyses for both island‐groups and the Mantel test for Florida populations indicated that there were no significant relationships between genetic and geographic distances. The lack of relationship between genetic similarity and geographic distance can be explained in a variety of ways without invoking nonrandom processes. Two examples include (i) no gene flow among populations and (ii) panmixia.

Although the Mantel test for populations within Belize resulted in a marginally significant value (*p* = .0457), the result of this test in and of itself cannot be used as prima facie evidence of IBD. Significant results of Mantel tests provide evidence of spatial correlation as a rejection of an open gene flow model (Meirmans, 2012). However, a Mantel test may result in an erroneously significant *p*‐value because it cannot discriminate among a host of alternative spatially structured models, such as IBD and geographic clustering (Meirmans, 2012; Guillot & Rousset, 2013). Because of this, and because the linear regression analysis was nonsignificant, there is insufficient evidence to conclude that the Belizean populations fit the IBD model.

### 4.5 Life history traits

The dispersal capabilities of this species, whether by active swimming or drifting in ocean currents, directly impact gene flow within and among island‐groups. The dispersive ability of *A. stipes*, however, has never been explicitly studied, although it is likely that it exhibits life history traits that are homologous to closely related atherinid species (Takemura, Sado, Maekawa, & Kimura, 2004; Francisco et al., 2009; Gotoh et al., 2011; Mazlan et al., 2012). These atherinid species display various ecological characteristics that result in reduced dispersal capabilities, including adhesive demersal eggs that attach to vegetation, a short larval stage with well‐developed larvae, and a strict association with coastal environments (Takemura et al., 2004; Francisco et al., 2009; Gotoh et al., 2011). The eggs of *A. stipes* from Belize and Florida in our samples had filaments (B Chernoff, pers. obs.).

The pelagic larval state of marine fishes can be rather difficult to track due to their small size and the spatial scale of distribution (Mora & Sale, 2003; Cowen, Gawarkiewicz, Pineda, Thorrold, & Werner, 2007). However, as Shulman and Bermingham (1995) concluded, ocean current patterns and length of the pelagic larval phase may have the greatest influence on marine fish dispersal and population connectivity. Although little is known about the dispersal abilities of *A. stipes*, the ocean currents flow to the north and east from Belize (Shulman & Bermingham, 1995) and may help explain the presence of Belizean haplotypes in four individuals from the southern Florida Keys.

Additionally, high levels of larval dispersal among populations may diminish signals of IBD at smaller spatial scales over time (D'Aloia et al., 2014). The lack of barriers to larval dispersal may explain why geographic distance did not correlate with the amount of genetic differentiation at the island‐group scale. Despite this difficulty, it is important to determine the dispersal potential of *A. stipes* in order to elucidate the biological mechanisms that facilitate gene flow.

## 5 CONCLUSION AND FUTURE WORK

The Belizean and Floridian island‐groups were found to be highly divergent from each other. Within island‐groups, populations exhibited high numbers of haplotypes and differed significantly from one another though there was no or insufficient evidence for IBD. Populations within both island‐groups were characterized by high percentages of shared haplotypes and high percentages of rare but “unique” haplotypes. Two potential hypotheses were discussed that treat the common haplotypes as evidence of gene flow among populations. They differ by the amount of gene flow required and by the interpretation of the rare “unique” haplotypes. While our data cannot reject either hypothesis, the lack of shared haplotypes among neighboring populations and the very high percentages of rare haplotypes may favor the hypothesis requiring lower gene flow among populations. The results of neutrality tests and the haplotype network provide strong evidence that Floridian populations have undergone recent expansion following a bottleneck. The pattern of evolution among Belizean populations is less clear due to the complexity of their haplotype network and to the limitation of some of the population genetic statistics to discern more historic population patterns. How the population genetic structures can result from dispersive life history traits of *A. stipes* is unclear. Unfortunately, the dispersal capabilities between and within island‐groups, in addition to accurate population size estimates, of *A. stipes* are currently unknown.

The close association of *A. stipes* to mangrove habitats throughout the Caribbean presents an ideal opportunity to examine the potential influences of habitat fragmentation on intraspecific genetic structuring within and among island‐groups. Mangrove ecosystems are critical habitats because they serve as nurseries, feeding grounds, and shelters for many marine organisms (Laegdsgaard & Johnson, 2001). The destruction of mangrove communities has occurred at a staggering rate, as approximately one‐third of the world's mangroves have disappeared over the last 60 years (Alongi, 2002; Hamilton & Casey, 2016). This was caused by a variety of anthropogenic factors, such as aquaculture, agriculture, industrial and residential development, forestry uses, and recreational planning (Ellison & Farnsworth, 1996; Valiela, Bowen, & York, 2001). Regardless of whether the mangroves are removed, local disturbances that increase sedimentation and water turbidity negatively affect foraging species, such as *A. stipes*, which rely heavily on visual cues to search for food in the water column (Thresher, 1983; Vaslet, Bouchon‐Navaro, Charrier, et al., 2010; Vaslet, Bouchon‐Navaro, Louis, et al., 2010). Furthermore, the alteration and elimination of mangrove habitats may contribute to population isolation and extinction.

As this study only analyzed a single mitochondrial gene, there were limitations on the conclusions that we were able to make. It is important that future studies examine additional genomic regions and perform morphological analyses in order to reveal details of gene flow between and within island‐groups. Sampling from other populations of *A. stipes* at other island localities throughout the Caribbean will help to illuminate diversification patterns. While this study presents the first genetic analysis of Hardhead silverside populations, a comprehensive examination of the phylogeography throughout the Caribbean is critical for future conservation and management of *A. stipes*.

## CONFLICT OF INTEREST

None declared.

## AUTHORS CONTRIBUTION

All authors equally contributed to the preparation of this manuscript.

## ENDNOTE

1 We acknowledge that if sample sizes were to get very large, the probability of finding a population‐specific or “unique” haplotype in another population increases in proportion to the degree of gene flow among populations.

## APPENDIX A

### A.1

**Table A1:** Island population details

Location	Location code	Sample size	Sample ID number	Voucher ID	Collection date	Latitude	Longitude
Belize							
Carrie Bow Cay	B‐CB	31	A01–A31	USNM 349215, C001–C030	7/17/2014	16.802189	−88.081992
South Water Cay	B‐SW	29	A32–A43, A45–A61	C031–C042, C044–C060	7/17/2014, 7/18/2014	16.814498	−88.082381
Twin Cays	B‐TC	1	A44	C043	7/18/2014	16.828901	−88.103603
Tobacco range	B‐TR	29	A62–A90	C061–C089	7/19/2014	16.881713	−88.094155
Blue Range Cays	B‐BR	26	A91–A116	C090–C115	7/20/2014	16.822198	−88.148925
Cat Cay	B‐CC	30	A117–A146	C116–C145	7/21/2014	16.65671	−88.180175
Stewart Cay	B‐SC	29	A147–175	C146–C174	7/22/2014	16.770558	−88.163134
Florida Keys							
Key Largo	F‐KL	29	A177–A206	F001–F030	8/18/2015	25.131944	−80.400556
Plantation Key	F‐PK	30	A207–A236	F031–F060	8/18/2015	24.989611	−80.552644
Marathon Key	F‐MK	9	A237–A245	F061–F069	8/18/2015	24.726078	−81.013431
Key West	F‐KW	30	A246–A275	F069–F099	8/19/2015	24.556681	−81.77295
Summerland Key	F‐SK	28	A276–A305	F100–F129	8/19/2015	24.684317	−81.44425
Spanish Harbor Key	F‐SH	28	A306–A335	F130–F159	8/20/2015	24.654633	−81.301508
Marathon Key‐Aviation Road	F‐MA	30	A336–A365	F160–F189	8/20/2015	24.724089	−81.065275
Key Biscayne	F‐KB	30	A366–A395	F190–F219	8/21/2015	25.727603	−80.156078

This table contains the name of each sampled island population, the location code, the number of individuals sampled, the sample ID number, the specimen voucher ID number, the date of collection, and the latitude and longitude of each sample site. The sample sizes reflect successfully sequenced individuals minus the four highly differentiated Florida fish.

**Table A2:** Neutrality tests

Location code	Sample size	Tajima's D	Fu's *F*_S_	Fu and Li's D*	Fu and Li's F*
Belize					
B‐CB	31	−1.35341	−7.769***	−3.10127*	−2.98953*
B‐SW	29	−1.19021	−2.779	−2.68751*	−2.60012*
B‐TC	1	–	–	–	–
B‐TR	29	−1.16416	−4.469*	−1.31937	−1.49241
B‐BR	26	−1.4015	−6.653**	−2.3408	−2.40097
B‐CC	30	−1.16663	−4.239*	−2.82277*	−2.69938*
B‐SC	29	−0.81383	−1.51	−0.3179	−0.55725
Florida Keys					
F‐KL	29	−2.09958**	−8.96824***	−2.88909*	−3.09979*
F‐PK	30	−2.32245***	−14.5953***	−3.60522**	−3.78146**
F‐MK	9	−1.435	−3.71002**	−1.36983	−1.55045
F‐KW	30	−1.6786*	−4.98768***	−1.39132	−1.73711
F‐SK	28	−2.16246**	−14.1063***	−3.3281**	−3.47596**
F‐SH	28	−2.09697**	−13.017***	−3.90939**	−3.91803**
F‐MA	30	−2.50648***	−15.7964***	−3.85979**	−4.03119**
F‐KB	30	−2.15792**	−14.6372***	−2.21797	−2.58691

The values and significance of Tajima's D, Fu's *F*_S_, Fu and Li's D*, and Fu and Li's F* neutrality statistics generated in Arlequin.

Significance is indicated as follows: **p *<* *.05, ***p *<* *.02, ****p *<* *.001. The sample sizes reflect successfully sequenced individuals minus the four highly differentiated Florida fish.
